# Flow cytometric characterization of freshly isolated and culture expanded human synovial cell populations in patients with chronic arthritis

**DOI:** 10.1186/ar2916

**Published:** 2010-01-27

**Authors:** Kristel B Van Landuyt, Elena A Jones, Dennis McGonagle, Frank P Luyten, Rik J Lories

**Affiliations:** 1 Laboratory for Skeletal Development and Joint Disorders, Division of Rheumatology, Katholieke Universiteit Leuven, Herestraat 49, Leuven, B3000, Belgium; 2 Academic Unit of Musculoskeletal Disease, Leeds Institute of Molecular Medicine, University of Leeds, St James's University Hospital, Beckett Street, Leeds, LS9 7TF, UK

## Abstract

**Introduction:**

The synovium is a major target tissue in chronic arthritis and is intensively studied at the cellular and molecular level. The aim of this study was to develop flow cytometry for the quantitative analysis of synovial cell populations pre and post culture and to characterize mesenchymal cell populations residing in the inflammatory synovium.

**Methods:**

Knee synovium biopsies from 39 patients with chronic arthritis and from 15 controls were treated in a short, standardized tissue digestion procedure. Stored, thawed digests were routinely analyzed with flow cytometry including live-dead staining and use of the markers CD45, CD3, CD14, CD20, CD34, CD73, CD105, CD90, CD146, CD163 and HLA-DR to distinguish inflammatory and stromal cells. The influence of the digestion method on the detection of the different surface markers was studied separately. In addition, we studied the presence of a specific cell population hypothesized to be mesenchymal stem cells (MSC) based on the CD271 marker. Cell expansion cultures were set up and a MSC-related surface marker profile in passages 3 and 6 was obtained. Immunohistochemistry for CD34 and von Willebrand factor (vWF) was done to obtain additional data on synovium vascularity.

**Results:**

The cell yield and viability normalized to tissue weight were significantly higher in inflammatory arthritis than in controls. Within the hematopoietic CD45-positive populations, we found no differences in relative amounts of macrophages, T-lymphocytes and B-lymphocytes between patient groups. Within the CD45-negative cells, more CD34-positive cells were seen in controls than in arthritis patients. In arthritis samples, a small CD271 positive population was detected. Culture expanded cells were found to fulfill the multipotent mesenchymal stromal cell marker profile, except for CD34 negativity. Detection of peripheral blood macrophage and B-cell markers was decreased after enzymatic exposure and mechanical forces, respectively, but stromal markers were not affected.

**Conclusions:**

Flow cytometry can distinguish synovial cell populations in tissue digests. The preparation method can influence the detection levels of macrophage and B-cell populations. However, stromal cell markers seem not affected and quantification is possible, supporting flow cytometry tissue analysis as a tool to study these cell populations in arthritis.

## Introduction

Inflammation, loss of synovial homeostasis and progressive synovial-mediated joint destruction are critical features of inflammatory arthritis. Not surprisingly, the synovium has been a preferred tissue for the investigation of cellular and molecular mechanisms in arthritic diseases such as rheumatoid arthritis (RA) and spondyloarthritis (SpA). Historically, synovial tissue analysis was carried out in tissue obtained during joint replacement surgery but advances in needle arthroscopy with increased access to small synovial biopsies has facilitated the study of synovitis at the cellular and molecular levels. Currently, synovial tissue analysis is used in clinical trials, in translational science studies and even in routine clinical practice [1].

Immunohistochemistry has become a standard method of analysis for the detection and quantification of synovial cell populations [2]. With this method, both semi-quantitative and quantitative digital image analyses have been proposed as tools to compare samples [1,2]. However, immunohistochemistry has a number of drawbacks. By its two-dimensional character, reconstruction of the total biopsy content is a laborious task, while each tissue slice analyzed provides information about a rather limited cell number. The interfacing steps between visible signals and quantitative digital data are not fully automated and therefore quite labour intensive and specialized. Performing double staining is possible, but multicolor staining is very difficult to achieve. Further, the technique is certainly not devoid of variability between centers with variations in tissue preservation, fixation, and staining. Even if under perfect circumstances specific molecular signals can be detected and localized reliably, this information represents the endpoint of investigation because the cells cannot be studied further with respect to their function.

Another method for studying synovial cells is the *in vitro *expansion of synovium-derived, plastic-adherent cell populations, mostly fibroblast-like cells. Molecular and functional characteristics obtained with these cells can be counted as circumstantial evidence for mechanisms involved in disease pathogenesis [3-5]. However, this approach will probably not deliver the key information about the mechanisms by which these stromal cells modulate tissue responses *in vivo*. Selection of plastic adherent cells is typically used to study cell phenotype and function, but this further stresses the question to what extent manipulations influence cell behavior in comparison with the original *in vivo *situation. Multiple variables are inherent to the technique: polyclonal versus clonal culture, use of bovine products or not, and passage kinetics can all influence the cell phenotype which is finally studied.

Flow cytometry is a robust and well-established technique that has found wide application in cellular characterization and could overcome many of the limitations associated with immunohistochemistry and tissue culture. However, it has thus far surprisingly little application in joint-derived tissue in the study of arthritis. Flow cytometry is known as an excellent phenotyping tool, because multiple molecules, surface markers as well as intracellular proteins, can be detected in individual cells [6]. Also, it is appreciated for its rapid, automated quantification leading to the evaluation of rare cells. Finally, sorting of the cells allows their further use in functional *in vitro *or *in vivo *assays. Hence, flow cytometry is an attractive application for synovial tissue analysis.

Flow cytometry has been previously used to study non-cultured synovial cell suspensions, focusing either on lymphocyte and macrophage cell populations [7-9] or on the characterization of mesenchymal cells [10] or both [11]. We wanted to use known basic markers for stromal cells [12] as well as additional promising markers such as CD146 (melanoma cell adhesion molecule [13] and CD271 (low-affinity nerve growth factor receptor) [14-17] that were recently associated with specific multipotent mesenchymal cell populations, as a first step to explore different populations of synovium-derived cells. We analyzed the cellular composition of fresh synovial tissue digests with flow cytometry to develop a standard operating procedure. Given that solid tissue enzymatic disaggregation is known to cleave certain membrane markers we also evaluated how this affected the results obtained. Herein, we propose a robust protocol that permits the study of fresh human synovial cell populations with widespread application in translational research and we demonstrate that this method is useful to distinguish specific stromal cells.

## Materials and methods

### Patient and control materials

Fifteen control patients suffering from knee pain and planning to undergo orthopedic knee arthroscopy, were recruited into the study. Diagnostic data were recorded during the arthroscopy. A total of 39 arthritis patients, suffering from inflammatory knee synovitis and referred for needle arthroscopy by their rheumatologist, were included in this study. Twelve arthritis patients fulfilled the revised American College of Rheumatology criteria for RA [18]. In 24 patients a diagnosis of SpA was made with the patients fulfilling the European Spondyloarthropathy Study Group criteria [19] or in case of psoriatic arthritis, the ClASsification of Psoriatic ARthritis study group criteria [20]. In three patients with chronic synovitis (more than six weeks' duration), no definitive diagnosis could be made. In orthopedic patients but not in arthritis patients, the blood flow was controlled during the arthroscopy procedure by the application of a tourniquet above the knee joint. Peripheral blood was obtained from healthy volunteers. Human cord blood (not fulfilling clinical cryopreservation criteria) was a gift from the Hematology Department. The procedures were approved by the Local Ethics Committee (University Hospitals K.U. Leuven) and written informed consent was obtained from each patient.

### Isolation and cryopreservation of synovial cells

In control patients, three large biopsies (median total weight 342 mg, quartiles 258 to 370 mg) were collected during orthopedic knee arthroscopy by the surgeon while in arthritic patients, four smaller biopsies (median total weight 45 mg, quartiles 24 to 83 mg) were obtained blindly during a single port needle arthroscopy. After collection, tissue was rinsed in Dulbecco's PBS (DPBS; Lonza, Verviers, Belgium) and finely minced with a scalpel and a needle. A two-step procedure was used for the digestion. First, hyaluronidase from bovine testes (SigmaAldrich, Bornem, Belgium), was dissolved in DMEM supplemented with 1% antibiotics/antimycotics + 1% sodium pyruvate (SP) (all from Invitrogen, Merelbeke, Belgium) + 0.1% albumin bovine fraction V (Serva, Heidelberg, Germany), to obtain a hyaluronidase solution of 1000 units/ml (approximately 0.1%), and filtered with a 0.22 μm filter (Millipore, Brussels, Belgium). For orthopedic samples, a double amount (4000 units/sample) of active enzyme compared with arthritis samples (2000 units/sample) was used due to the larger size of the biopsies. The minced tissue was dropped in the solution, weighed, and subsequently incubated for 15 minutes at 37°C with continuous rotation at 16 to 20 revolutions/minute (rpm). A second enzyme solution consisted of 1000 units/sample (arthritis samples) or 2000 units/sample (orthopedic samples) of collagenase type IV from *Clostridium histolyticum *(Invitrogen, Merelbeke, Belgium). This solution was added to the tubes containing the first solution and the tissue, the final concentration of collagenase being 400 units/ml (approximately 0.2%). After two hours of further digestion with rotation, cells were centrifuged, washed and sieved through a nylon 70 μm cell strainer (BD Biosciences, Erembodegem, Belgium). Cells were centrifuged and resuspended in growth medium being DMEM supplemented with 1% antibiotics/antimycotics, 1% SP and 10% fetal bovine serum (FBS) (Bio Whittaker, Verviers, Belgium). Cells were counted in a Bürker chamber after staining with trypan blue (Sigma-Aldrich, Bornem, Belgium). The suspension was either immediately seeded in cell culture (see below) or was gradually mixed at 1:1 with freezing medium being DMEM supplemented with 20% FBS and 20% dimethylsulfoxide (DMSO; Sigma-Aldrich, Bornem, Belgium). In three arthritis patients, 20% of the digest was seeded in cell culture while the rest was frozen in order to compare the digest with the cultured cells from the same patient. Cells were frozen gradually (in an isopropanol freezing container) to -80°C overnight and transferred afterwards to liquid nitrogen.

### Staining of frozen digests

For flow cytometry staining, antibodies conjugated with fluoro-isothiocyanate (FITC), phycoerythrin (PE) or allophycocyanin (APC) were used. CD45-FITC (clone HI30), CD19-PE (clone HIB19), CD73-PE (clone AD2), CD90-PE (clone 5E10), CD146-PE (clone P1H12), CD163-PE (clone GHI/61), CD271-PE (clone C40/1457), human leucocyte antigen (HLA) DR-PE (clone G46-6), CD3-APC (clone UCHT1), CD34-APC (clone 581), and isotype controls immunoglobulin (Ig)G_1_-FITC, IgG_1_-PE, IgG_1_-APC were purchased from BD Biosciences (Erembodegem, Belgium). CD20-PE (clone LT20), CD14-APC (clone TUK4) and IgG_2a_-APC were purchased from Miltenyi-Biotech (Utrecht, the Netherlands). CD105-APC (endoglin, clone 166707) was from R&D systems (Minneapolis, MN, USA). 7-amino-actinomycin (7-AAD) (Calbiochem, San Diego, CA, USA) was reconstituted with PBS/DMSO 4:1 to a concentration of 200 μg/ml. For washes, incubation and acquisition of the data, cells were suspended in incubation buffer (DPBS + 2% FBS + 1 mM ethylenediaminetetraacetic acid (EDTA)). Data were acquired on a FACSCanto cytometer (BD Biosciences, Erembodegem, Belgium) and analysis of the data was performed using BD FACSDiva 5.0 software (BD Biosciences, Erembodegem, Belgium). The synovial cells were thawed quickly, suspended in 5 ml of DMEM/10% FBS and centrifuged. Cells were resuspended in incubation buffer. For each sample, there were five different staining tubes with a minimum of 10,000 and a maximum of 500,000 cells per tube. For each patient, three or four different triple stainings were performed, constantly including CD45 combined with two other markers. This doublet could be CD20 with CD3, CD14 with CD163, or CD34 with CD73, CD90, CD146, CD271 or HLA-DR. For each patient, isotype control stainings were matched to these combinations. Per tube, 5 to 20 μl of antibody was applied directly on the pellet (depending on the cell number and the manufacturers' recommendations, with a minimum of 5 μl per tube), followed by an incubation of 45 minutes at 4°C in the dark. Cells were washed with incubation buffer, spun down and resuspended in 250 μl of incubation buffer. 7-AAD solution was added (to a final concentration of 2.5 μg/ml), followed by incubation during 10 minutes on ice.

### Cell culture

For five control samples and five samples from RA patients, all isolated cells were seeded in a plastic culture dish (Greiner, Wemmel, Belgium). In addition, for three samples from undifferentiated arthritis and SpA, part of the obtained cells were seeded in order to compare the digest with the cultured cells for each patient. Cells were seeded on a fixed surface area, 10 cm^2 ^for arthritis samples and 75 cm^2 ^for control samples. Cells were allowed to attach during four to five days before replacing the growth medium being DMEM supplemented with 1% antibiotics/antimycotics, 1% SP, and 10% FBS. After 10 to 14 days, when cells were confluent, they were rinsed with DPBS, enzymatically harvested with TrypLE express (Invitrogen, Merelbeke, Belgium), washed, counted, and replated at a density of approximately 5000 cells/cm^2^. Further passages were performed upon confluence. Cells were expanded up to passage six for control and RA patients, and up to passage one or two (culture period around four weeks) for three additional arthritis patients.

### Flow cytometry staining of cultured cells

The choice of the antibodies used was based on the minimal surface marker panel proposed by the International Society of Cellular Therapy [12]. CD45-FITC, CD34-FITC (clone 581), CD14-PE (clone MΦP9), CD73-PE, CD79a-PE (clone HM47), CD90-PE, HLA-DR-PE, IgG1-FITC, IgG1-PE, IgG2a-PE were from BD Biosciences (Erembodegem, Belgium). CD20-PE was from Miltenyi (Utrecht, the Netherlands) and CD105-FITC (clone 166707) was from R&D systems (Minneapolis, MN, USA). Upon confluence, cells from one 75 cm^2 ^flask were harvested, washed and counted. They were kept on ice and suspended in incubation buffer (DPBS + 2% FBS + 1 mM EDTA). After centrifugation and aspiration of supernatant, 5 or 10 μl (depending on the cell number per tube) of the antibodies were applied directly onto the pellet. Only one antibody was used per tube to minimize signal compensation problems with the strong autofluorescence signal emerging from cells in higher passage numbers. Cells were incubated at 4°C for 30 to 45 minutes. Cells were washed, resuspended, and data were acquired and analyzed. For three other patient samples, cultured cells (P1 or P2) were only stained with CD271-PE and with CD34-APC and their controls, here also including a double staining.

### Measurement of enzymatic and mechanical effects

Peripheral blood mononuclear cells (PBMCs) were isolated from venous blood by density centrifugation with Lymphoprep (Axis Shield, Oslo, Norway). One part of the cells was immediately used for flow cytometry, other parts were subjected to the mechanical and enzymatic compounds of the synovial digestion procedure. For enzymatic exposure, 6 to 48 × 10^6 ^PBMCs were treated with 4000 units hyaluronidase and 2000 units collagenase in the same concentrations as used for the digestion of synovium. For mechanical exposure, PBMCs were subjected to rotation at different velocities (8 to 20 rpm). Cells were centrifuged, and subsequently counted and used for flow cytometry analysis. In two experiments, a part of the exposed cells were allowed to recover for two hours at room temperature before staining.

For CD34 expression, cord blood mononuclear cells (CB-MNCs) were freshly isolated by density centrifugation. Partial enrichment for CD34-positive fraction was performed with an EasySep CD34 selection kit using the clone QBend10 (Stem Cell Technologies, Grenoble, France). Subsequently, cells were divided into two conditions: one part was immediately stained for CD34-APC (clone 581), the other part was exposed to the combined enzymatic and mechanical (8 rpm) treatment.

For control on expression of CD73, CD90, and CD105, human synovium-derived cells from RA patients were cultured in plastic culture flasks for three or five passages. The cells were harvested, washed and counted. Part of the cells was exposed to the combined enzymatic and mechanical (8 rpm) treatment, the other part was directly stained. A similar protocol was used to test for the expression of CD146, but here, fibroblasts were not used but human osteosarcoma cells (gift from Dr. J. Klein-Nulend from the Free University of Amsterdam). CD146 is known as an endothelial marker in healthy tissues, but it is also a multifaceted molecule that can act as a promoter or suppressor of cancer [21].

Cells were stained with the tested antibodies and in conditions similar to staining of the fresh digests, but on cultured cells, no double or triple stainings were used due to autofluorescence of these cells. For PBMCs and CB-MNCs, approximately 700,000 cells were used per tube in the stainings.

### Immunohistochemistry of the synovial tissue

Six synovial biopsies per patient were fixed in 4% formaldehyde overnight, and transferred to methanol the next day. After embedding in paraffin, serial sections (5 μm) were prepared on silanated slides. Quenching was performed twice with 3% H_2_O_2 _for 10 minutes. Sections were washed twice in PBS with 0.1% TritonX. After a blocking step of 30 minutes with 1:5 diluted donkey serum (Chemicon, Temecula, CA, USA), sections were incubated overnight at 4°C with the following monoclonal antibodies diluted in PBS/Triton: von Willebrand Factor (vWF) (mouse anti-human IgG1κ clone F8/86 purchased from Dako, Heverlee, Belgium) at 5 μg/ml and CD34 (mouse anti-human IgG1κ clone 581 purchased from BD Biosciences (Erembodegem, Belgium) at 10 μg/ml. For controls, a mouse isotype antibody (Chemicon, Temecula, CA, USA) was used at the same concentration. For the detection of vWF, a horseradish peroxidase (HRP)-conjugated goat anti-mouse IgG (Jackson, Suffolk, UK) was used at 4 μg/ml and incubated during 30 minutes. After washing steps, this was detected by an enzymatic reaction with 3,3'-diaminobenzidine in chromogen solution (Dako, Heverlee, Belgium). For the detection of CD34, a donkey anti-mouse biotin-conjugated IgG-B (Santa Cruz, Heidelberg, Germany) was used at 2 and 4 μg/ml (on consecutive sections), followed by washing steps and incubation with an Avidin and Biotinylated HRP Macromolecular Complex (Vector laboratories, Burlingame CA, USA) before final color reaction with 3,3'-diaminobenzidine. Evaluation of the staining was performed with a numerical qualitative score. Score 1 was given to sections with the lowest amount of positive cells, 2 to a median amount, and 3 to the highest amount of positive cells.

### Statistical analysis

The Mann-Whitney U Test was used to detect differences between groups. Statistical significance was defined as *P *< 0.05. The Spearman Rank Correlation Test was used to detect correlation.

## Results

### Patient and tissue characteristics

**Figure 1:**
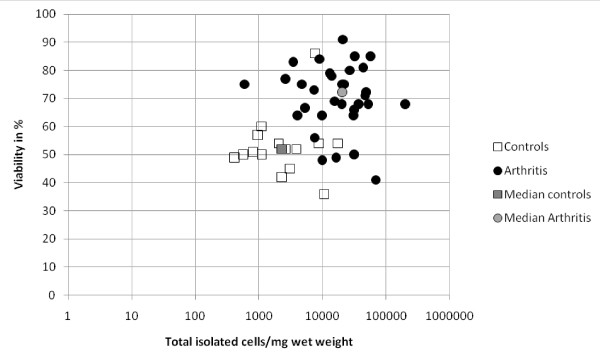
**Microscopic evaluation of synovial cell yield and viability directly after isolation**. Viability was counted as cells positive for trypan blue.

Control patients (seven males/eight females) had a median age of 44 years. The reason for orthopedic arthroscopy was knee pain due to cartilage lesions (five patients), meniscal lesions (three patients), combined cartilage-meniscal injury (five patients), cruciate ligament repair (one patient), and removal of a non-resorbed intra-articular screw (one patient). Characteristics of the patients from the arthritis group with established diagnosis are shown in Table 1. The median cell yield after digestion was 2286 cells/mg in the control group vs. 20,222 cells/mg in the arthritis group (*P *< 0.001; Figure 1). The viability of the cells by trypan blue exclusion was statistically different between both groups (median 52% vs. 72%; *P *< 0.001). There was no correlation between cell yield and viability for both groups.

**Table 1:** Characteristics of arthritis patients

	Rheumatoid arthritis (n = 12)	Spondyloarthritis (n = 24)
Age (years)	43 (37.8-51.2)	37.5 (31-47.2)
Male/female	5/7	9/15
Disease duration (months)	33 (4.25-114)	7.5 (3.75-24)
CRP (mg/l)	39.7 (7.4-69.2)	10.5 (2.9-32.2)
ESR (mm/h)	41 (21-60.7)	25 (16.5-51)
WBC SF (cells/μl)	12,100 (9450-15500)	6900 (3050-10100)
DMARDs in use (by number of patients)	no (7), hydroxychloroquine and sulphasalazine (1), methotrexate (1), leflunomide (1), anti-TNF (2)	no (18), sulphasalazine (2), methotrexate (3), leflunomide (1)
RF (% positive patients)	55	11
Anti-CCP (% positive patients)	70	19

Numbers indicate median with first and third quartiles unless otherwise indicated.

Anti-CCP = anti-cyclic citrullinated protein antibodies; CRP = C-reactive protein; DMARDs = disease-modifying anti-rheumatic drugs; ESR = erythrocyte sedimentation rate; RF = rheumatoid factor; WBC SF = white blood cell count in synovial fluid.

### Flow cytometry of frozen digests

**Figure 2:**
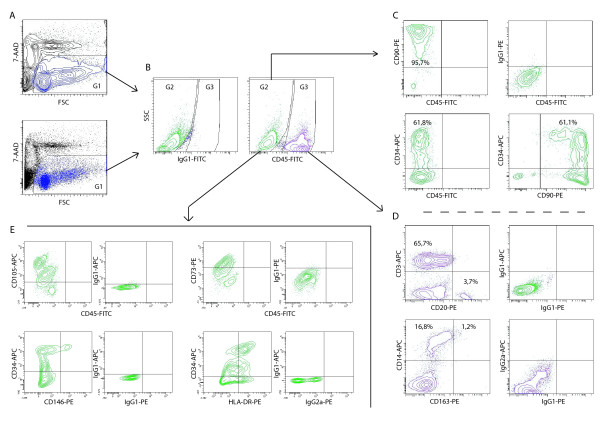
**Analysis of total synovium-derived cell population with four-color flow cytometry: representative sample**. Cells are stained with 7-aminoactinomycin (7-AAD), CD45- fluoro-isothiocyanate (FITC), one phycoerythrin (PE)-conjugated antibody and one allophycocyanin (APC)-conjugated antibody or their isotypes. **(a) **Dye exclusion of debris and dead cells in forward scatter (FSC)/FL3 in contour plot (top) and dot plot (bottom). G1 gates for live cells. **(b) **Subgating of CD45-negative stromal (G2) and CD45-positive (G3) hematopoietic cell populations based on isotype staining with IgG1-FITC and cell granularity (side scatter). **(c) **Quantification of CD34-expression and CD90-expression within the stromal fraction. The gating for CD90-positive cells was based on IgG1-PE specifically measured on CD45-negative cells. **(d) **Quantification of CD14, CD3, and CD20-expression within G3. The gating for CD20-positive cells was performed on IgG1-PE measured on total cells. **(e) **Plots showing CD105-APC, CD73-PE, CD146-PE, and human leucocyte antigen (HLA)-DR-PE staining in the stromal fraction with their appropriate isotype controls.

Detection of the synovium-derived cells on the flow cytometer is based on two critical elements. Firstly, viability staining excludes dead or apoptotic cells and debris defining a gate based on forward scatter and on staining for propidium iodide (PI) [11] or 7-AAD (Figure 2a). Secondly, staining for CD45 separates cells of non-hematopoietic origin from hematopoietic cells in the FL1 channel [11] (Figure 2b). Other cell markers were analyzed in the FL-3 (PE) and FL-4 (APC) channel. Within non-hematopoietic cells, a majority of cells was CD90-positive. This population comprises fibroblasts and endothelial cells [22]. CD34 was positive in a subset of CD90-positive cells and nearly all CD34-positive cells also stained for CD90, albeit not brightly (Figure 2c). Within hematopoietic cells, populations representing T-lymphocytes (CD3+), B-lymphocytes (CD20+) and monocytes (CD14+) could be recognized (Figure 2d). The staining for CD163, a marker for alternatively activated macrophages in the synovium [23] was not sufficiently discriminative for quantification. Within the stromal compartment (CD45-negative cells), large populations expressing CD73 (median 81.3%, n = 8) and CD105 (median 86%, n = 3) and smaller populations expressing HLA-DR (median 36.5%, n = 6) and CD146 (median 5.6%, n = 8), also in part CD34-positive, were seen (Figure 2e).

**Figure 3:**
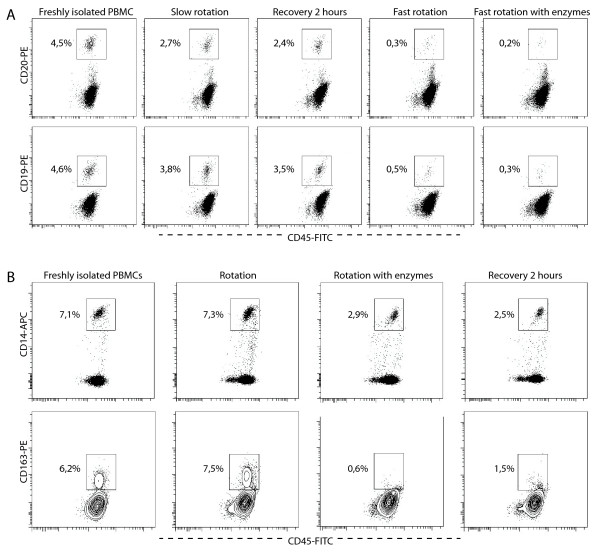
**Influence of the isolation procedure on the detection of B-cell and macrophage markers: representative experiments**. Peripheral blood mononuclear cells (PBMCs) from healthy volunteers stained with 7-aminoactinomycin (7-AAD), CD45- fluoro-isothiocyanate (FITC) and **(a, top panel) **CD20- phycoerythrin (PE) or **(a, lower panel) **CD19-PE, **(b, top panel) **CD14- allophycocyanin (APC) or **(b, lower panel) **CD163-PE. Shown events were gated 7-AAD-negative and CD45-positive. Positivity for cell markers is shown in percentage of gated events. **(a) **Influence on B-cell markers. Slow rotation: 8 rpm. Fast rotation: 16 to 20 rpm. **(b) **Influence on monocyte/macrophage markers.

**Figure 4:**
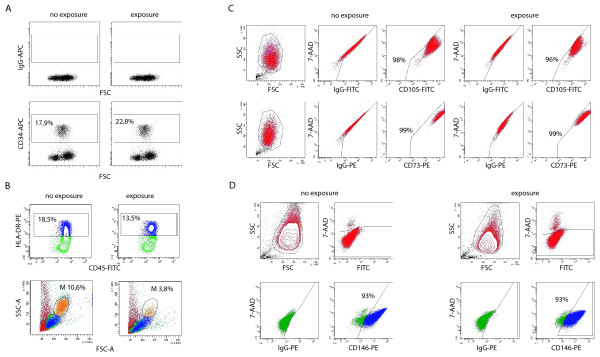
**Influence of the isolation procedure on the detection of stromal markers**. **(a) **Dot plot showing CD34- allophycocyanin (APC) and isotype staining of viable CD45-positive CD34-enriched cord blood mononuclear cells (CB-MNCs) before and after exposure to the digestion method. **(b) **Plots showing (top panel) human leucocyte antigen (HLA)-DR- phycoerythrin (PE) staining and (lower panel) forward scatter (FSC)/side scatter (SSC) of viable, CD45-positive peripheral blood mononuclear cells (PBMCs) before and after exposure to the digestion method. **(c) **Dot plots showing CD73-PE, CD105- fluoro-isothiocyanate (FITC) and isotype stainings of cultured passage five synovium-derived mesenchymal stem cells. **(d) **Plots showing CD146-PE and isotype staining in viable (negative for 7-aminoactinomycin (7-AAD) in peridinin chlorophyll protein channel) human osteosarcoma cells before and after exposure to the digestion method.

Before quantifying the different populations, we tested whether tissue digestion might influence the detection of immune cell markers, by exposing PBMCs to the procedure or its components and subsequently staining for CD3, CD14, CD163, and CD20 (see Table 2 for experiment set-up). B-cell and to a lesser extent macrophage markers appeared to be sensitive to the procedure. In specific experiments, the expression of CD19 and CD20 disappeared almost completely after rotation at 16 to 20 rpm, while slower rotation (8 rpm) could partially prevent this effect (Figure 3a). Expression of CD14 and CD163 was decreased by enzyme exposure but not further decreased by rotation (Figure 3b). Markers of each cell type decreased in a parallel way, suggesting cell death to be the cause. On the forward scatter (FSC)/side scatter (SSC) plots, we confirmed the disappearance of macrophages/monocytes. Also, the relative proportion of CD3-expression was increased, suggesting decrease of the other populations. We also tested whether expression of stromal markers was affected by exposure to the complete digestion procedure in cultured cells. Expression of CD34 in CD34-enriched CB-MNCs was not affected (Figure 4a). Expression of HLA-DR was affected to the same extent as the dying of monocytes in total PBMCs (Figure 4b). Expression of CD73 and CD105 (Figure 4c) and CD90 (data not shown, off-scale fluorescence) in cultured synovium-derived cells was not affected by the procedure, as was the expression of CD146 in human osteosarcoma cells (Figure 4d).

**Table 2:** Influence of the digestion method on composition of peripheral blood mononuclear cells

	Collagenase units/10^6 ^cells	Condition	CD3 %	CD14 %	CD163 %	CD20 %
Donor 1	40	fresh	73.3	5.4	5.3	7.2
		exposed	82.8	3.4	0.3	0.9
Donor 2	80	fresh	77.2	7.4	7.4	6.4
		exposed	86.2	4.6	1.2	0.6
Donor 3	340	fresh	53	16.4	19.4	4.8
		exposed	62.3	12	6.8	0.7

### Quantification and detecting differences between patient groups

**Figure 5:**
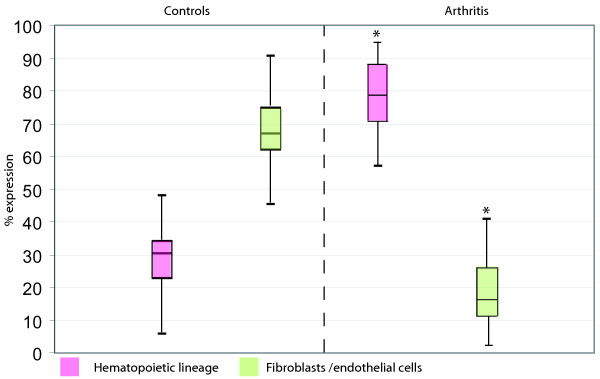
**Expression level of CD45 in control samples versus arthritis samples in percentage of viable cells**. Boxes = median, 25^th ^and 75^th ^percentiles. Whiskers = minimum and maximum values. *P < 0.05.

**Figure 6:**
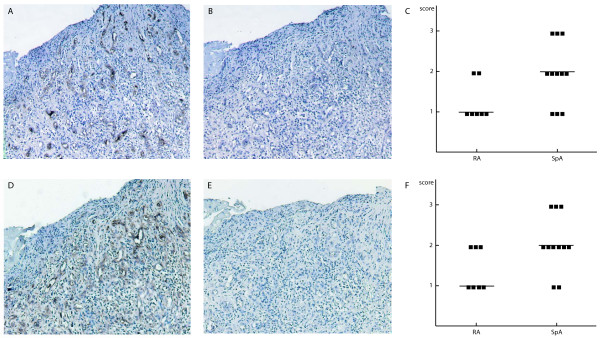
**Semi-quantitative assessment of vascularization in synovial tissue by immunohistochemistry**. Magnification 100×. **(a to c) **von Willebrand Factor staining. **(a) **Positive endothelial cells are detected. **(b) **Isotype control. **(c) **Positive (sub)endothelial cells and fibroblasts are detected. **(d to f) **CD34 staining. **(e) **Isotype control. **(c and f) **Scores in different patient groups.

As expected, biopsies from patients with inflammatory arthritis contained a significantly larger proportion of hematopoietic cells than the orthopedic controls (median CD45-positivity 79.6% vs. 30%, *P *< 0.0001) and a smaller proportion of non-hematopoietic cells than the controls (median CD45-negative cells 16.9% vs. 67%, *P *< 0.0001; Figure 5). Other results from the quantification are depicted in Table 3. No differences were detected concerning the CD45-positive subpopulations. Within CD45-negative cells, the CD90-positive population was not different between RA and SpA samples. The CD34-positive population contains endothelial cells [24]. CD34-expression was higher in the control samples than in arthritis samples (*P *= 0.034). CD34-expression was also higher in SpA than in RA, however, not statistically significant (*P *= 0.089). By immunohistochemistry for CD34 and vWF, median scores for CD34 were two in the SpA group and one in the RA group (*P *= 0.053). CD34 was positive in (sub)endothelium and also in certain stromal elements (Figure 6). There were no significant correlations between stromal/hematopoietic cell composition of the synovial digests and the inflammatory status of the patient as assessed by C-reactive protein level, erythrocyte sedimentation rate or the white blood cell count of the synovial fluid.

**Table 3:** Quantification of surface marker expression in synovial digests

		RA (n = 7)		SpA (n = 12)	
			
		median (%)	quartiles	median (%)	quartiles
CD45-positive	CD3	72.7	57.9-76.7	66.6	58.6-79.6
	CD14	14	7.8-22.3	12.9	6.9-16.6
	CD20	4.2	1.9-6.8	4.1	2.1-6.3
CD45-negative	CD34	34.1	26.1-45.2	53.8	44.4-61.9
	CD90	82.1	80.1-95.3	84.9	71.8-90

Numbers indicate percentage of positive cells counted within each group of CD45-positive or CD45-negative cells.

RA = rheumatoid arthritis; SpA = spondyloarthritis.

### Marker expression in cultured cells

The direct *ex vivo *cell characterization involves the comparison with standard plastic-adherent, cultured cells. Therefore, cultured cells from five control patients and five RA patients were also tested for their surface marker profile, largely with the same antibodies as those used in the study of the digests. Cell population doubling was linear over time between passage one and six [see Additional file 1]. The cells showed no expression of CD45, CD20/CD79a, or HLA-DR, but were positive for CD73, CD90, and CD105 in passage three and six (Table 4). The plots are shown in Additional file 2. Data were variable for the marker CD34. Most cultures showed expression of this marker which did not decrease during passaging. This result was tested and confirmed by PCR or quantitative PCR in a series of 10 other cell cultures (data not shown). Expression of CD34 was lower in RA cultures than in control cultures in parallel with the fresh digests, but this was not statistically significant. Low levels of CD14 were detected in most cultures but tended to decrease during passaging.

**Table 4:** Surface marker phenotype of cultured synovium-derived cells

	Control (n = 5)		RA (n = 5)	
		
Passage	P3	P6	P3	P6
**Marker**				
				
CD45	0	0	0	0
CD14	9 (4-35)	10 (4-11)	22 (19-27)	17 (13-18)
CD34	30 (24-72)	60 (57-70)	25 (12-30)	32 (9-41)
CD20/CD79a	0	0	0	0
HLA-DR	0	0	0	0
CD73	100	100	100	100
CD90	99	99	99	100
CD105	98	99	98	97

Numbers indicate percentage of positive cells calculated as medians (first and third quartiles between brackets).

RA = rheumatoid arthritis.

### Detection of CD271 as a specific marker for a stromal subpopulation in the synovium and in expanded cells

**Figure 7:**
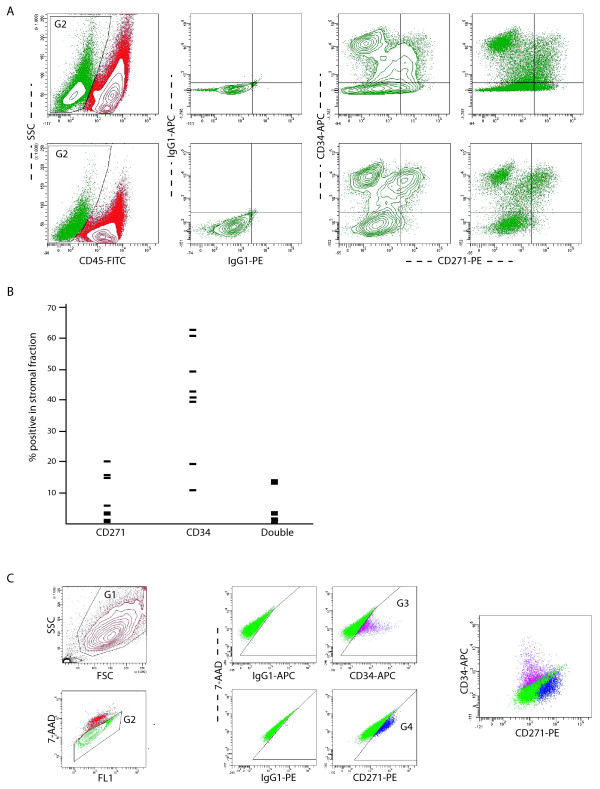
**Detection and quantification of CD271 and CD34 in digests and cultured cells**. **(a) **Plots of two representative samples. The CD45-negative fraction is gated in G1. Within this fraction, a portion of cells is positive for CD271 or CD34. Double positive cells are sometimes well clustered (lower plot). To be uniform, the quantification is performed using quadrant analysis. **(b) **Data points representing the expression levels in percentage positivity on the cell surface detected in the fresh digests. **(c) **Plots of cultured cells after staining. Cells are gated according to scatter properties (G1), viability (G2) and the signal for the markers is compared with the isotype controls. No double positive cells are recognized here. 7-AAD = 7-aminoactinomycin; APC = allophycocyanin; FITC = fluoro-isothiocyanate; FSC = forward scatter; PE = phycoerythrin; SSC = side scatter.

CD271 has been associated with bone marrow-derived mesenchymal progenitor cells but does not appear to be present on expanded mesenchymal progenitor cells from the synovium [14,16,17,25]. We therefore used this molecule as an example to see whether CD271-positive cells are present in the synovium. In a set of eight patients, staining for CD34 was combined with CD271. The plots are illustrated in Figure 7a. CD271 was expressed in most of the samples (Figure 7b), with a median value of 4.7% of the stromal compartment, and 3.1% of the stromal cells were both CD271 and CD34 positive. In contrast, expression in synovium-derived fibroblastic cells cultured for one month was tested in three patients of whom digest data were also available. A certain expression of CD271 was maintained but cells positive for both CD271 and CD34 were not longer detected (Table 5).

**Table 5:** Comparing marker expression in the digests and cultures from the same samples

	Digested			Cultured		
		
	CD271	CD34	double	CD271	CD34	double
Patient 1	0.2	11	0	11	1	0
Patient 2	6	40	3	10	12	0
Patient 3	16	41	13	4	0.7	0

Numbers indicate percentage of positive cells counted within the CD45-negative gating.

## Discussion

This study used flow cytometry to provide a novel method for the quantification and characterization of human synovium cell populations and showed that stromal and non-stromal populations could be detected and quantified. However, enzymatic synovial disaggregation may be associated with changes in cell surface marker expression, which needs to be considered when using this approach. Given the robust nature of cytometry and the ability to simultaneously evaluate multiple cell populations, this method has considerable potential for the evaluation of synovial tissue.

Fresh tissue digests with good quality isolated cells open the way to many downstream research applications in synovial biology such as cell sorting [11], magnetic separation, extraction of RNA or proteins, *in vivo *transfer models and the prospective purification of putative stem cell populations. The isolation procedure described here has a number of advantages. Its short timeline may limit changes in the activation status of some cells. Also, quality of the tissue digest can be checked by simple microscopic cell counting with dye viability exclusion, an assessment which was sufficient to discriminate between our two different biopsy types. The higher cell yield found in arthritis versus control synovium is in line with the known features of hypertrophy and immune cell infiltration. The higher viability using arthritis samples cannot be explained by differences in enzyme concentration because this was the same for both types of samples. The mechanical disruption step could have less impact in the case of the more loose inflamed arthritis tissue, and as such provoke less cell death as in the non-inflamed tissue.

However, some concerns remain and are relevant for the cell isolation process. First, the fact that this isolation procedure is short does not infer that cell survival or signaling activity would be left unchanged. Nevertheless, far less artifacts are expected than is the case after cell culture. The influence of the procedure can be tested separately for each marker as we illustrated. Second, reproducibility of digest analysis between centers can play an important role. It is clear that for optimal results and complete reproducibility between centers, which is needed in clinical trials, procedures should be agreed upon at start and standardized across centers.

Procedures for cell isolation from tumors [26], gut mucosa [27], liver [28], and cartilage [29] were reported to affect the final flow cytometry readout. We detected influences on macrophage and B-cell marker expression relying on mimicking experiments with PBMC suspensions. The relative expression of macrophage markers and B-cell markers were decreased by exposure to the enzyme cocktail or to the rotation process, respectively. Neither expression was restored after two hours. CD14 function has been described to be sensitive to enzymatic cleavage [30] and receptor shedding. In our case, however, selective cell death seems to be the major cause because the population diminished equally on FSC/SSC plots. Also, the parallel decrease of two phenotype markers on each cell type suggests cell death. On the other hand, artefacts may be overemphasized in the mimicking experiments because the normal substrates for the digesting enzymes are not present in these conditions. Importantly, the expression of markers used for the stromal compartment was not decreased by the digestion. This makes the method more valid for the quantification of stromal subpopulations than infiltrating subpopulations. If the method is used to study certain hematopoietic subsets, further study of the conditions is necessary.

In the case of CD163 and CD20 expression, we would have expected differences between RA and SpA patients considering previous immunohistochemical observations [31,32], which was not confirmed. In the stromal fraction, we found a higher proportion of CD34-positive cells in samples from control patients than from arthritis patients. So, in non-inflammatory synovium, more CD34-positive cells are recovered in relation to the other stromal populations than in the inflammatory synovium, which contains a bulk of hypertrophic synoviocytes in the villi. Also, a trend towards a higher proportion of CD34-positive cells was seen in SpA samples compared with RA samples. The latter is in line with macroscopic and microscopic observations of higher synovial vascularity in these patients. As CD34 is not specific for blood vessels in synovium [33] and could indicate other stromal cells, we performed immunohistochemical assessment combining CD34 and vWF. This confirmed the trend towards higher vascularity in the SpA group.

The surface marker profile of directly isolated cell populations was compared with that of plastic-adherence selected cultured synovium-derived cells. We expected the latter to have a conventional mesenchymal stem cell profile. Unexpectedly, the expression of CD34 was present in the majority of cultures, albeit that the signal was not as strong as for the markers CD73, CD105, and CD90. The control samples show slightly more CD34-expression than the RA cultures. The CD34 positivity reflects the expression on fibroblasts and not the survival of endothelial cells or endothelial progenitor cells in the cultures [33]. True endothelial cells are rare in digests and unlikely to be maintained in large quantities in culture, in conditions not supporting their growth. Instead, CD34 has been a previously unnoticed signal in routinely cultured synovial cells through its weakness on a high fluorescence background. In contrast to frozen digests, in flow cytometry of cultured cells, this background excludes the use of multicolor staining in order to avoid severe compensation problems, at least when using advanced passage numbers. The baseline fluorescence of fluorescence-minus-one and isotype controls was accepted, because a lowering of the photomultiplier tube voltage settings would have decreased the signal-to-noise ratio in the detectors under the acceptable range. As sensitivity was as such preserved, the weaker signal was still detectable. The results were reproduced with two different antibody clones and three different fluorochromes. However, in bone-marrow-derived cultured cells, the CD34-signal was absent, as also described before [34].

To challenge our method, we tested the presence of CD271-positive cells in the synovium. This marker appears to be absent from expanded synovial progenitor cells [25] but is present on bone marrow-derived progenitor cells and hence could be an *in vivo *marker of mesenchymal stem cells [35]. Our data indicate that a specific subpopulation of CD271-positive cells is present in inflamed synovium but that these cells are not at all or barely found in expanded cells populations from the same patients. This confirms the potential value of direct analysis to obtain direct information about stromal cell populations that could play a role in arthritis.

In general, the technical hurdles for flow cytometry in fresh digests of synovium are not larger than those in cultured synovial cells. Cell culture will be useful to further study functioning of the cells. However, it will be necessary to perform cytometry of functionally important molecules in the digests. It adds to the technique of immunohistochemistry that cells still can be stimulated, sorted, or cultured and it is useful in particular for the stromal subpopulations.

## Conclusions

This study reports on the use of enzymatic and cytometric techniques for the characterization of synovial cell populations. The major synovial tissue composition can be quantified and classical stromal markers remain unaffected by the digesting procedure. Further refinements are needed for optimization of cell viability and defining marker combinations specific for mesenchymal subpopulations. These findings will help open up the way for direct and functional studies of synovial cell populations *ex vivo *in humans.

## Abbreviations

7-AAD: 7-aminoactinomycin; APC: allophycocyanin; CB-MNCs: cord blood mononuclear cells; DMEM: Dulbecco's Modified Eagle Medium; DMSO: dimethylsulfoxide; DPBS: Dulbecco's phosphate-buffered saline; EDTA: ethylenediaminetetraacetic acid; FBS: fetal bovine serum; FITC: fluoro-isothiocyanate; FSC: forward scatter; HLA: human leucocyte antigen; HRP: horseradish peroxidase; IgG: immunoglobulin G; PBMC: peripheral blood mononuclear cells; PBS: phosphate-buffered saline; PCR: polymerase chain reaction; PE: phycoerythrin; PI: propidium iodide; RA: rheumatoid arthritis; SSC: side scatter; SP: sodium pyruvate; SpA: spondyloarthritis; vWF: von Willebrand Factor.

## Competing interests

The authors declare that they have no competing interests.

## Authors' contributions

KV, RL and FL participated in the design of the study. Experiments in Leuven were performed by KV. Training and preliminary experiments were performed in Leeds by EJ and DM. Analysis of the data was performed by KV, RL and EJ. The manuscript was drafted by KV and was commented and revised by RL, EJ, FL and DM. All authors read and approved the final manuscript.

## Supplementary Material

Additional file 1 PDF file containing growth curves of cell cultures derived from **(a) **control and **(b) **inflamed synovium starting from passage one to six. ORT = cells derived from control samples; REU = cells derived from RA patients with active knee arthritis. Population doublings during each passage were calculated as the logarithm to two of the fold increase of cells (being harvested cells divided by seeded cells). Click here for file

Additional file 2 PDF file containing plots showing the surface marker profile of cultured synovium-derived cells: representative sample. **(a) **Dot plots depicts the marker of interest in x-axis with 7-aminoactinomycin (7-AAD) staining in y-axis. **(b) **Dot plots depicting the controls used. The upper row shows the used isotype controls, with not much aspecific binding. The lower row shows cells only stained with 7-AAD ('fluorescence-minus-one' control). Click here for file
