# Supplementary figures and images for: Flow cytometric characterization of freshly isolated and culture expanded human synovial cell populations in patients with chronic arthritis
Source: Arthritis Res Ther. 2010 Jan 27;12(1):R15. doi: 10.1186/ar2916 (PMC2875643; doi:10.1186/ar2916)

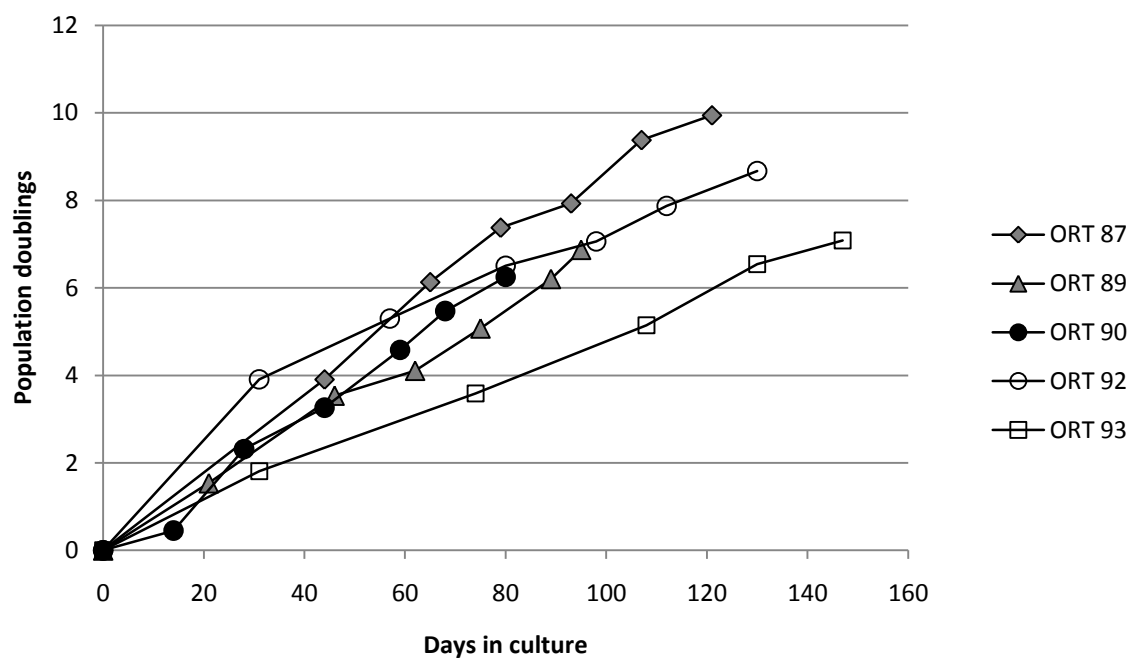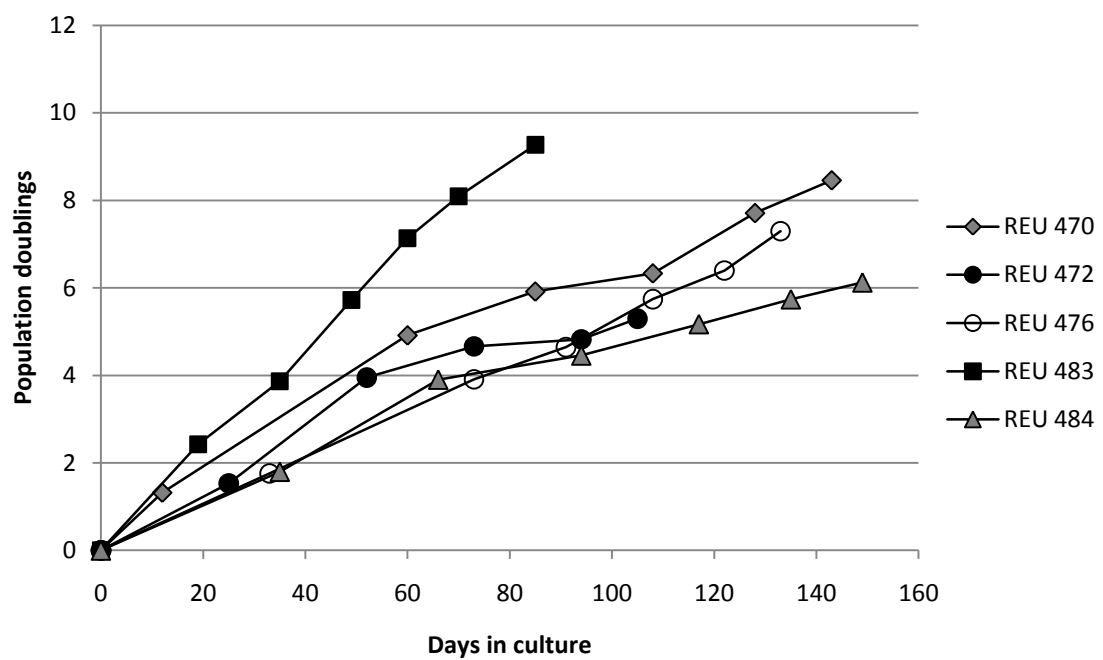

Supplement: Additional file 1 — PDF file containing growth curves of cell cultures derived from (a) control and (b) inflamed synovium starting from passage one to six. ORT = cells derived from control samples; REU = cells derived from RA patients with active knee arthritis. Population doublings during each passage were calculated as the logarithm to two of the fold increase of cells (being harvested cells divided by seeded cells). [file ar2916-S1.pdf]

A

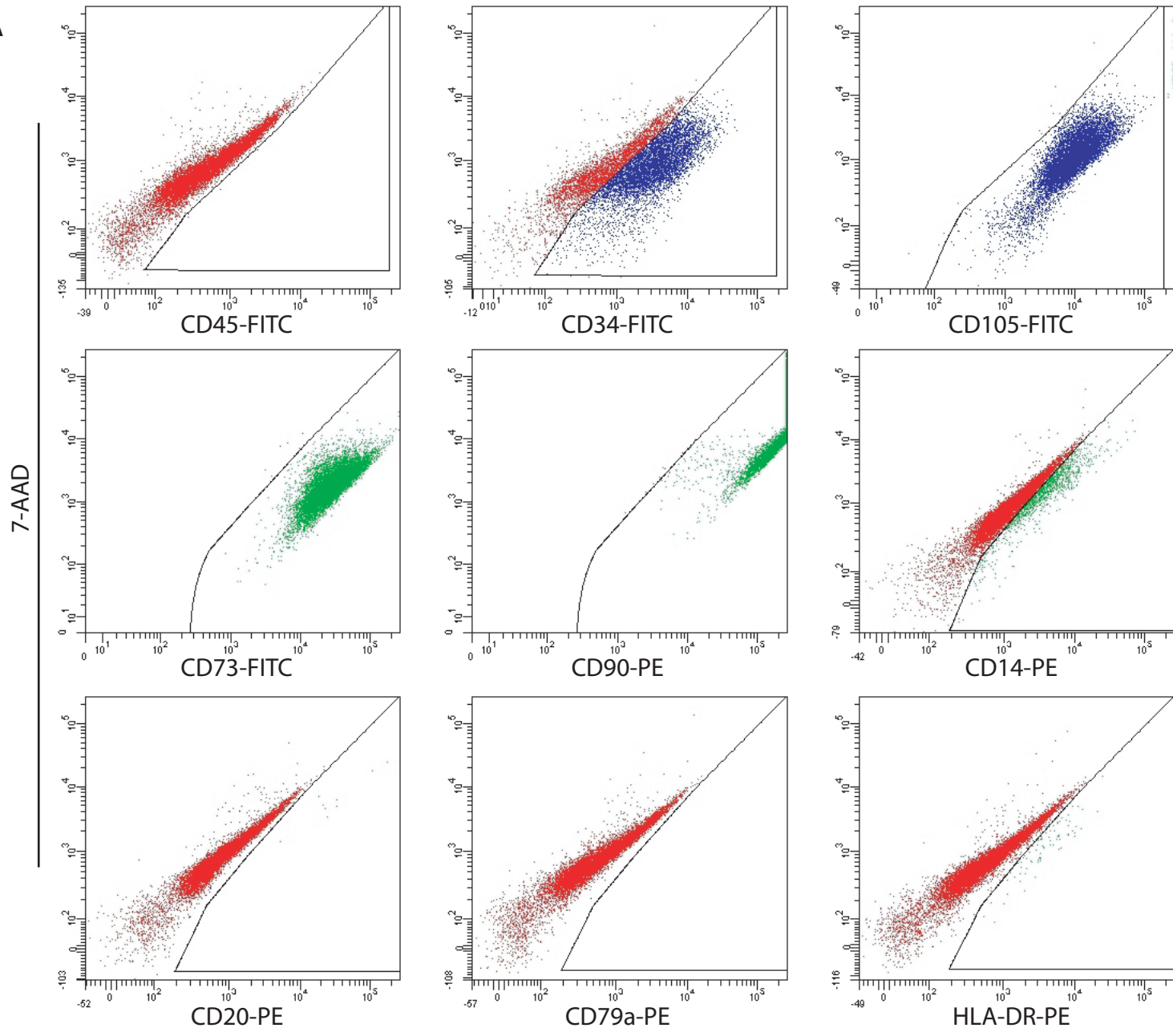

B

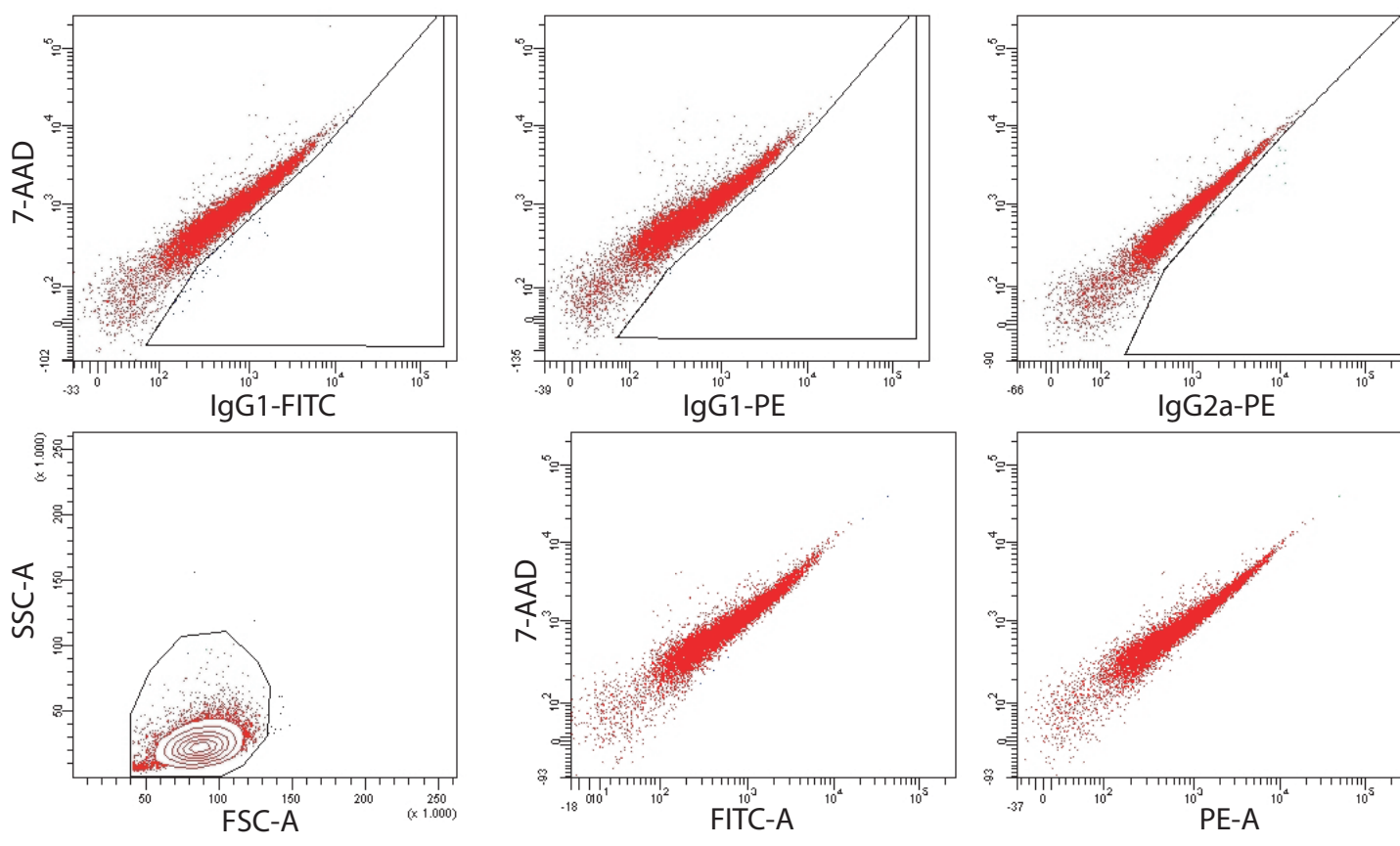

Supplement: Additional file 2 — PDF file containing plots showing the surface marker profile of cultured synovium-derived cells: representative sample. (a) Dot plots depicts the marker of interest in x-axis with 7-aminoactinomycin (7-AAD) staining in y-axis. (b) Dot plots depicting the controls used. The upper row shows the used isotype controls, with not much aspecific binding. The lower row shows cells only stained with 7-AAD ('fluorescence-minus-one' control). [file ar2916-S2.pdf]
